# WALANT–Epinephrine injection may lead to short term, reversible episodes of critical oxygen saturation in the fingertips

**DOI:** 10.1007/s00402-020-03744-5

**Published:** 2021-01-23

**Authors:** P. Moog, M. Dozan, J. Betzl, I. Sukhova, H. Kükrek, K. Megerle

**Affiliations:** 1 Division of Hand Surgery, Klinikum Rechts der Isar, Technical University of Munich, Munich, Germany; 2 Klinik und Poliklinik für Plastische Chirurgie und Handchirurgie, Klinikum Rechts der Isar der, Technischen Universität München, Ismaninger Str. 22, 81675 München, Germany

**Keywords:** WALANT, Wide awake surgery, Epinephrine, Finger necrosis, Digital nerve block anesthesia

## Abstract

**Introduction:**

Although the WALANT technique’s long-term safeness has been demonstrated in many studies, there are only few data investigating its short-term effects on tissue perfusion and oxygen levels. It was hypothesized that, temporarily, critical levels of tissue perfusion may occur.

**Methods:**

Seventeen patients, who were scheduled for different procedures in WALANT technique, were injected with 5–7 ml of 1% Articain containing 1:200,000 epinephrine at the finger base. Capillary-venous oxygen saturation, hemoglobin volume in the capillaries, and relative blood flow in the fingertips were recorded once per second by white light spectrometry and laser Doppler flowmetry before, during and after injection for an average of 32 min.

**Results:**

Clinically, no persistent tissue malperfusion was observed, and there were no postoperative complications. Capillary-venous oxygen saturation was reduced by ≥ 30% in seven patients. Critical levels of oxygen saturation were detected in four patients during 13 intervals, each lasting for 132.5 s on average. Oxygen saturation returned to noncritical values in all patients by the end of the observation period. Blood flow in the fingertips was reduced by more than 30% in nine patients, but no critical levels were observed, as with the hemoglobin. Three patients demonstrated a reactive increase in blood flow of more than 30% after injection.

**Conclusions:**

Injection of tumescent local anesthesia containing epinephrine into finger base may temporarily cause a substantial reduction in blood flow and lead to critical levels of oxygen saturation in the fingertips. However, this was fully reversible within minutes and does not cause long-term complications.

## Introduction

The injection of the tumescent local anesthetic lidocaine combined with epinephrine before finger surgery, known as the “Wide Awake Local Anesthesia No Tourniquet” (WALANT) technique, has been demonstrated to be safe from a long-term clinical perspective in many studies [1]. For quite some time, epinephrine was held responsible for finger necrosis, based on the case reports from before the 1950s [2, 3]. Meanwhile, authors have come to the agreement that the local anesthetic procaine was responsible for the described tissue necrosis and finger loss [2, 4]. In the early twenty-first century, the use of epinephrine as a chemical tourniquet in combination with local anesthetics began [5], and no cases of finger necrosis have been reported using lidocaine with epinephrine before 2000 [8]. Although its harmlessness has been clinically proven many times [6–9], textbooks and drug information continuously warn against its use in the acra [5, 10, 11], a dogma that has been passed on for generations [12]. Between 2014 and 2017, three case reports described total or partial finger necrosis after injection of lidocaine and epinephrine (1:100,000). Two resulted in the amputation of one or more finger end limbs, yet without having attempted reversal with phentolamine [13, 14]. In contrast, this proved effective in a third patient, whose postoperative finger ischemia could be reversed that way, saving the digit [15]. With regard to these case reports, one must consider, that in the time span they occurred, several thousand other operations in WALANT technique were performed successfully, without complications. Therefore, the overall risk of necrosis seems to be minimal while the benefits, such as a better intraoperative overview due to lower bleeding without tourniquet, the assessment of stability and gliding ability, predominately. Above all, cases that benefit in particular from WALANT technique are tendon surgery [1, 2, 4] and osteosynthesis [16, 17].

Nonetheless, there are only very few data about its short-term effects on tissue perfusion and oxygen supply after administering epinephrine [5, 18–20, 29]. Hence, this study’s aim was to investigate these by means of micro-lightguide spectrophotometry and laser Doppler flowmetry. Therein, this study focused on capillary-venous oxygen saturation (sO_2_), hemoglobin volume (rHb) in the capillaries, and relative blood flow in the fingertips. It was hypothesized that critical levels of tissue perfusion may occur after injection of Articain and epinephrine for WALANT.

## Materials and methods

### Ethical approval

This study was authorized by the ethics committee of our institution (137/18S). All patients had been scheduled for elective procedures regardless of participation in this study.

### Patients and methods

**Fig. 1:**
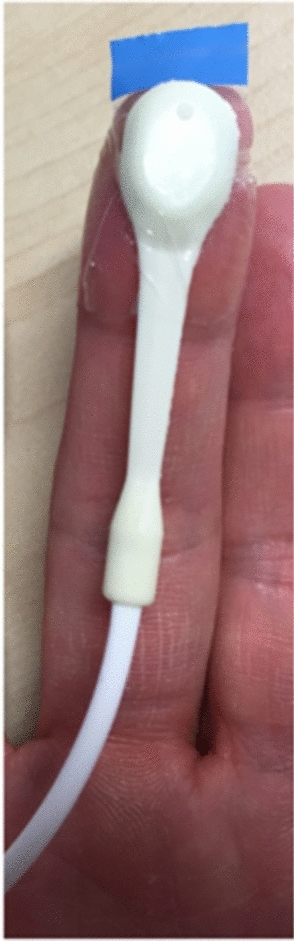
Correct position of the detection probe

In this prospective cohort study, the patients were recruited in our clinic from May through July 2019. Owing to technical difficulties, only 17 of initially 21 patients (9 women, 8 men) with a mean age of 55 years (range 23–79 years) could be included. They were scheduled for different procedures (15 patients: trigger finger release, 1 patient: mucoid cyst extirpation, 1 patient: removal of screw) using the WALANT technique, wherefore they were injected with 5–7 ml of 1% Articain containing 1:200,000 epinephrine subcutaneous at the palmar finger base, using a standardized technique as described by Harbison [21]. The patients were distracted by pressure outside the injection site and a 27G needle was introduced perpendicularly into the subcutaneous tissue. About 1 ml of solution was slowly injected and further infiltration was paused until the needle pain was gone. Then, the remaining solution was slowly injected without aiming for the digital nerves (Table 1). Patients with contraindications, such as allergic reactions, insufficient blood circulation (e.g., clinical signs, such as bluish discoloration, poor capillarization), Raynaud’s syndrome and glaucoma, were excluded. Tissue perfusion and blood flow were assessed using white light spectrometry and laser Doppler flowmetry (Oxygen to See, O2C, LEA, Germany, Fig. 1a).

**Table 1:** Included patients’ demographic data: Age, operated fingers, affected side, injection volume, and complications

Patient #	Age (years)	Gender (male/female)	Operated finger (D I–V)	Affected side (right/left)	Injection volume (ml)	Complications (yes/no)
1	62	Male	III	Right	6	No
2	58	Male	II	Right	5	No
3	57	Male	Lll	Left	6	No
4	81	Male	V	Right	5	No
5	55	Female	IV	Right	5	No
6	53	Female	I	Left	5	No
7	50	Female	I	Right	5	No
8	79	Female	III	Right	5	No
9	54	Female	IV	Right	5	No
10	60	Male	V	Right	7	No
11	25	Female	III	Left	5	No
12	73	Male	III	Left	5	No
13	48	Male	IV	Left	6	No
14	63	Female	Lll	Left	7	No
15	36	Male	V	Right	5	No
16	58	Female	III	Left	6	No
17	52	Female	I	Left	5	No

### Measurement procedure

The Oxygen to See (O2C) method provides continuous measurement and precise data collection by combining white-light spectrometry and laser Doppler flowmetry. Tissue is irradiated with light from a broad band and laser light source, while a sensor measures the remission produced by the tissue. White light remission (wavelength range of 500–850 nm) determines oxygen saturation (sO_2_) and relative hemoglobin (rHb) by calculating the amount of light absorbed by the hemoglobin, depending on its oxygen levels. Laser Doppler flowmetry measures blood flow using the Doppler shift of laser light waves (wavelength: 830 nm) caused by the remission from moving erythrocytes in the capillaries. The measurements’ penetration depths depend on the selected probe, with the maximum depth being 8 mm [22–24].

**Fig. 2:**
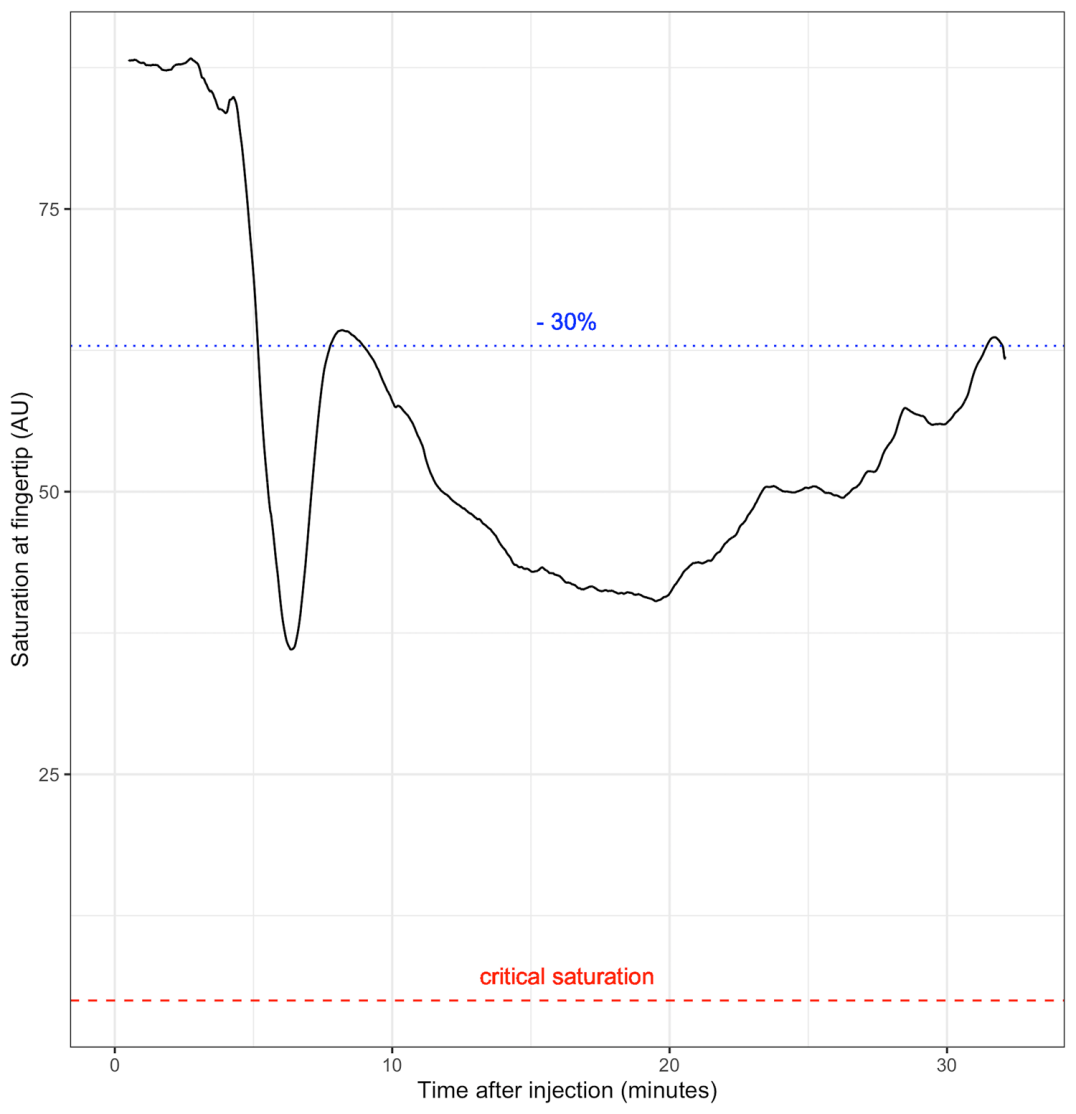
Example of oxygen saturation at the fingertip (AU) over time after injection. Saturation levels drop by more than 30%, but critical levels are never reached. Minimum saturation occurs after 380 s

Capillary-venous oxygen saturation (sO_2_ in %), hemoglobin volume in the capillaries (rHb in arbitrary units (AU)) and relative blood flow (in AU) in the fingertips were recorded once per second before, during and after injection of Articain and epinephrine, resulting in 43,956 data points. One patient’s (= patient 6 in Table 1) exemplary measurement curve for sO_2_ development after injection can be seen in Fig. 2.

Critical levels, irreversible or long-term values that would lead to tissue damage, were defined as < 10% for oxygen saturation (sO_2_), < 5 arbitrary units (AU) for blood flow and < 15 AU/ > 90 AU for hemoglobin volume (rHb), as suggested in the literature [23, 25, 26]. In addition, patient records were assessed for postoperative complications (Table 1).

### Evaluation of hemoglobin volume (rHb), oxygen saturation (sO_2_), and blood flow after injection

**Fig. 3:**
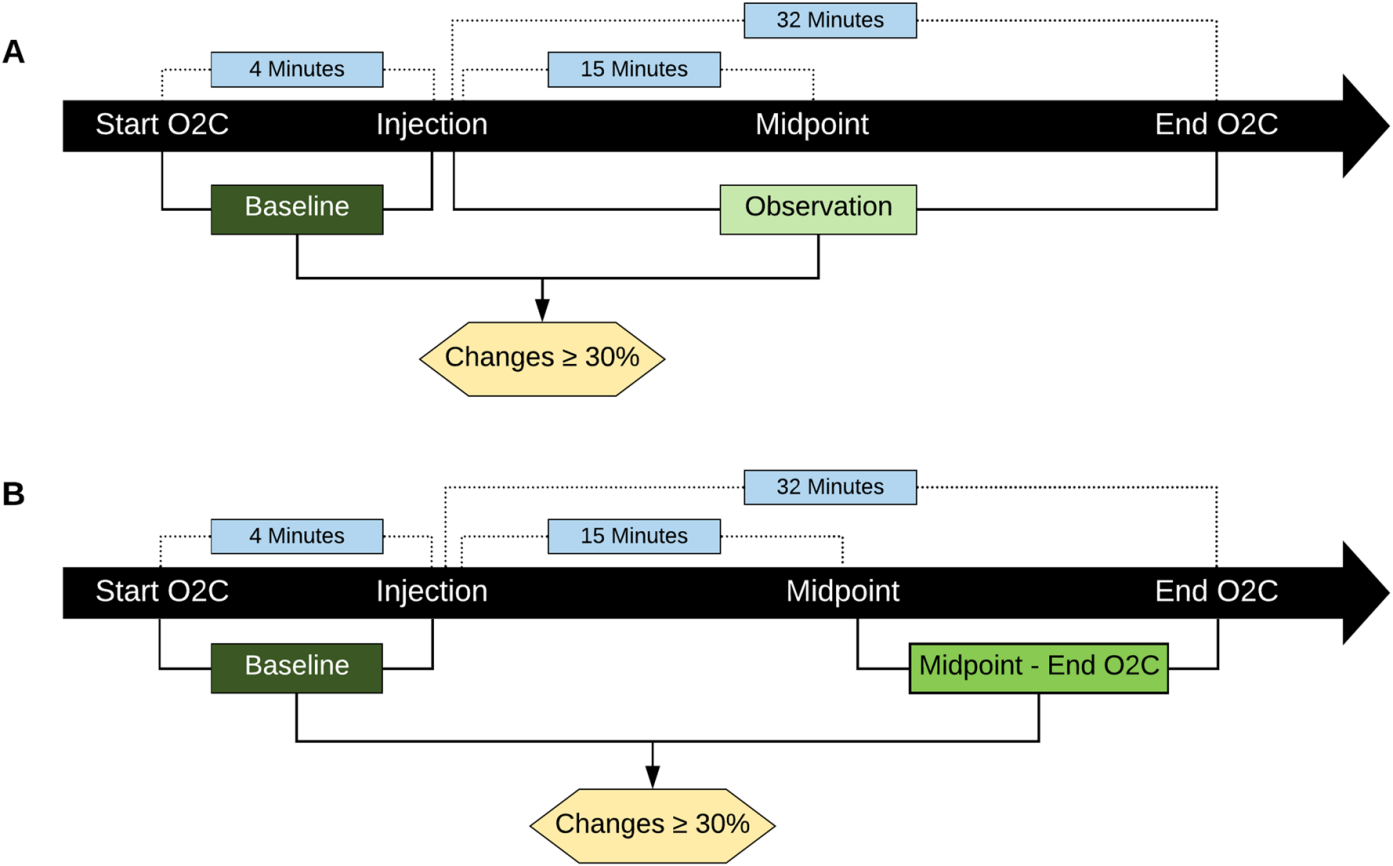
Sketch of the experimental set-up with mean times before (baseline) and after (observation values) injection and flow chart of the analysis procedure: **a** Baseline average and observation average were analyzed for changes in ≥ 30% (group A). **b** Baseline average and midpoint-end average were analyzed for changes in ≥ 30% (group B)

The phase from the start of the O2C measurement until injection of Articain and epinephrine was defined as “baseline”, which lasted for a mean time of 4 min (range 1–11 min, Fig. 3). The period after injection was called “observation”, lasting 32 min on average (range 29–79 min, Fig. 3). In addition, a third period was created, lasting from a midpoint (defined at 15 min after injection) until the end of the measurement (Fig. 3).

For data analysis, the single-point values of rHb, sO_2_ and blood flow, measured every second during the baseline and observation periods (see above), were averaged. In a second step, these average values were compared and analyzed for changes equal to or greater than 30% (Figs. 2, 3a) (= group A). The number of cases, in which such changes occurred, was determined.

Accordingly, the values measured for each of the parameters between the midpoint and the end of O2C were also averaged. These were then compared with the averaged values of the baseline period to evaluate whether the changes in this specific period were more pronounced than in the total observation phase. These results, too, were compared and analyzed for changes in equal to or greater than 30% (Fig. 3b) (= group B).

In a third step, the occurrence of critical levels of rHb (AU), sO_2_ (%) and blood flow (AU), as defined above, were examined for intervals of 60 s during the measurement by calculating rolling means. Their durations were recorded in seconds.

### Statistics

Owing to the small number of patients and their heterogeneous nature, only descriptive statistics were performed, but no hypotheses were tested.

## Results

### Development of rHb, sO_2_, and blood flow after injection

Averages between baseline values and observation period (Group A) demonstrated a drop of 30% or more in three patients for rHb levels, in seven patients in sO_2_ levels and in nine patients in flow levels. When assessing averages between baseline and midpoint end (Group B), reductions in 30% or more were found in three patients for rHb levels, in seven patients for sO_2_ levels, and seven patients for blood flow levels.

### Duration and proportion of critical oxygen saturation in the total observation time

The evaluation of the average values after injection showed a drop of the above-mentioned values, but without taking the previously defined critical values into account.

There were no critical values concerning rHb and blood flow throughout the entire investigation.

13 intervals of critical oxygen saturation with a mean duration of 132.5 s (range 4–482 s) were measured in four patients (Table 2). These intervals lasted a total of 1510 s and were most often found between 10 and 20 min after injection. At the end of the observation period, all patients demonstrated normal values.

**Table 2:** Occurrence of critical values per patient and their duration

	Time after injection (min)	Duration (s)
Patient 1	3	220
	16	91
	19	4
	19	7
	23	71
	26	177
Patient 3	2	138
	9	482
	21	64
Patient 8	5	345
Patient 13	1	44
	2	76
	15	15
	17	121

Generally, there were no allergic or other unexpected long-term reactions. Apart from the temporary occurrence of critical oxygen saturation values, we could not detect any permanent arterial ischemia or venous congestion.

## Discussion

The possibility to operate without a tourniquet is a major benefit for patients and surgeons [1, 4, 28]. The WALANT technique makes the surgeon independent of anesthesiologists and allows more flexibility in scheduling surgical treatments. WALANT represents an overall long-term, low-risk technique for every-day use. Apart from low general injection-related risks, such as infections and injuries to neurovascular structures, the contraindications to the injection of epinephrine, including allergic reactions, insufficient blood circulation, Raynaud’s syndrome and glaucoma must be considered. However, the combination of local anesthetic and epinephrine continues to be the topic of much discussion. Even though the application has been proven to be safe many times, textbooks and drug information warn against the use of local anesthetics with the addition of a vasoconstrictor in the acra, nose, ear, and penis.

In addition, there are recurring case reports of finger necrosis after injection for WALANT. For example, in 2014, eight hours after the excision of a skin tumor over a proximal interphalangeal (PIP) joint in WALANT anesthesia, a 16-year-old patient showed signs of ischemia, necessitating the amputation of the affected finger’s end limb. However, reversal of vasoconstriction with phentolamine had not been attempted and the authors could not rule out other causes of finger necrosis [14]. In 2017, an orthopedist in the United States injected lidocaine and 1:100,000 epinephrine for a trigger finger release and carpal tunnel decompression, leading to finger ischemia three hours postoperatively. This lasted for 14 h, until it could be reversed with phentolamine, saving the finger [15]. In the same year, in Canada, three fingers were injected for trigger finger release in WALANT technique. Phentolamine was not applied, leading to necrosis of two thirds of the index and middle finger end limbs and, subsequently, their amputation. The ring finger, whose tip also showed signs of necrosis, could be preserved [13].

Regarding the short-term effects of the WALANT technique on tissue perfusion and oxygen levels, there are very few data in scientific literature [5, 18–20, 29]. Therefore, it was examined tissue blood flow, capillary-venous oxygen saturation (sO_2_), and hemoglobin volume (rHb) in this study.

This study was able to perform complete and continuous monitoring of relevant post-capillary finger perfusion parameters after local injection for WALANT anesthesia. As the method of measurement used in this study is highly sensitive, however, not all critical values observed need to be considered clinically relevant. Even deep breathing can influence the parameters. Moreover, all values returned near baseline levels within the 30-min investigation period.

Many of our findings are in accordance with Altinyazar et al., who studied digital artery blood flow in 24 subjects, using color Doppler ultrasonography [5]. This was measured before digital blocking with lidocaine containing epinephrine, as well as 10, 60, and 90 min thereafter, resulting in a statistically significant decrease of blood flow rates. In addition, after 10 min of digital block, four patients showed no measurable blood flow, which, however, was restored within 60 or 90 min [5]. Although all values in all of our patients recovered within 30 min, the highest incidence of critical saturation was observed 10 to 20 min post-injection.

Concerning sO_2_, Sönmez et al. measured blood gas parameters before and 15 min after digital blocks, using lidocaine solutions with or without 1:80,000 epinephrine [18]. They reported that the sO_2_ first slightly increased, showing small reductions soon after. However, there were no significant differences whether the lidocaine was mixed with epinephrine or not [18]. In contrast, in this study, a continuous measurement is used and a more precise method of data collection, yielding similar results.

To determine changes in the blood flow, Sylaidis and Logan performed a quantitative study on 100 consecutive patients [19]. They measured the brachial and digital arterial systolic blood pressures before and after injection of 2% lidocaine and 1:80,000 epinephrine, finding that, although the digital blood pressures and the mean digital–brachial index decreased after the injections, this effect was completely reversible [19]. Schnabl et al. carried out a prospective, double-blinded, randomized study with 20 volunteers (80 fingers, without operation) [29]. The chronological course of changes in digit blood flow after injection of 0.75% ropivacaine and 1:1.000.000 epinephrine showed no significant changes with a following significant increase of skin perfusion (+ 66.6%) and prolonged pain reduction [29]. The blood flow measured at the fingertip was very variable in this study. On the one hand, this may be due to the sensitive measurement method; on the other hand, on the fingers’ extensor side, there remains sufficient regulation of the collateral vessels, so that blood flow in the fingertip remains relatively constant (unlike in the operating field).

Based on these data, however, no conclusions can be drawn as to how long one should wait after an injection, before starting the operation. McKee et al. found that after a subcutaneous infiltration of 5 ml lidocaine 1% with 1:100,000 epinephrine (0.01 mg/ml) into one arm, versus the same amount of lidocaine 1% without epinephrine into the other arm, the vasoconstrictive effect of lidocaine with epinephrine occurred after only 25 min (95% AI, 25.9 ± 5.1 min) [20]. In this study, a vasoconstrictive effect can be measured within a period of 10–20 min after injection, but normalizing within 30 min.

Despite the repeated demonstration of WALANT’s safety, phentolamine, an alpha-receptor blocker, may be used in the case of persistently low tissue perfusion. Injecting it as an antidote into the previously anesthetized area can remove the vasoconstrictive epinephrine effect (1 mg diluted in 1 ml saline) [2]. In our own practice, we have never had to use phentolamine over a course of more than 600 injections, as has D. Lalonde in the more than 2000 procedures he has performed using WALANT technique [27]. Nevertheless, like him, we always have the antidote available, to be prepared in the case of an emergency.

A limitation of this study is that the measurement is collected at the fingertip while the injection is done at the finger base. Presumably, the effect on the fingertip occurs after a delay, while the full effect on the finger base already exists, which leads to a time shift. Another limiting factor is the small number of cases and the variance of the injection volume and therefore the epinephrine related changes of the vasoconstrictive effect. Owing to the direct clinical preoperative application, the injection volume cannot always be completely standardized, since complete anesthesia must be ensured.

## Conclusion

Injection of tumescent local anesthesia containing epinephrine may cause a substantial reduction of blood flow and may lead to critical levels of oxygen saturation in the fingertips. However, these ischemic events seem to be short term, fully reversible within minutes and do not cause long-term complications. The WALANT technique can be considered safe for most patients.
